# Prediction of the World Health Organization Grade of rectal neuroendocrine tumors based on CT histogram analysis

**DOI:** 10.1002/cam4.3628

**Published:** 2020-12-01

**Authors:** Ping Liang, Chuou Xu, Fangqin Tan, Shichao Li, Mingzhen Chen, Daoyu Hu, Ihab Kamel, Yaqi Duan, Zhen Li

**Affiliations:** ^1^ Department of Radiology Tongji Hospital Tongji Medical College Huazhong University of Science and Technology Wuhan Hubei China; ^2^ Russell H. Morgan Department of Radiology and Radiological Science the Johns Hopkins Medical Institutions Baltimore MD USA; ^3^ Department of Pathology Tongji Medical College Huazhong University of Science and Technology Wuhan Hubei China

**Keywords:** computed tomography, histogram analysis, the grade rectal neuroendocrine tumors

## Abstract

**Objectives:**

To investigate the diagnostic value of contrast‐enhanced computed tomography (CECT) histogram analysis in predicting the World Health Organization (WHO) grade of rectal neuroendocrine tumors (R‐NETs).

**Materials and Methods:**

A total of 61 (35 G1, 12 G2, 10 G3, and 4 NECs) patients who underwent preoperative CECT and treated with surgery to be confirmed as R‐NETs were included in this study from January 2014 to May 2019. We depicted ROIs and measured the CECT texture parameters (mean, median, 10th, 25th, 75th, 90th percentiles, skewness, kurtosis, and entropy) from arterial phase (AP) and venous phase (VP) images by two radiologists. We calculated intraclass correlation coefficient (ICC) and compared the histogram parameters between low‐grade (G1) and higher grade (HG) (G2/G3/NECs) by applying appropriate statistical method. We obtained the optimal parameters to identify G1 from HG using receiver operating characteristic (ROC) curves.

**Results:**

The capability of AP and VP histogram parameters for differentiating G1 from HG was similar in several histogram parameters (mean, median, 10th, 25th, 75th, and 90th percentiles) (all *p* < 0.001). Skewness, kurtosis, and entropy on AP images showed no significant differences between G1 and HG (*p* = 0.853, 0.512, 0.557, respectively). Entropy on VP images was significantly different (*p* = 0.017) between G1 and HG, however, skewness and kurtosis showed no significant differences (*p* = 0.654, 0.172, respectively). ROC analysis showed a good predictive performance between G1 and HG, and the 75th (AP) generated the highest area under the curve (AUC = 0.871), followed by the 25th (AP), mean (VP), and median (VP) (AUC = 0.864). Combined the size of tumor and the 75th (AP) generated the highest AUC.

**Conclusions:**

CECT histogram parameters, including arterial and venous phases, can be used as excellent indicators for predicting G1 and HG of rectal neuroendocrine tumors, and the size of the tumor is also an important independent predictor.

## Graphical abstract

CECT histogram parameters, including arterial phase and venous phase, can be used as excellent indicators for predicting G1 and HG of rectal neuroendocrine tumors, and the size of the tumor is also an important independent predictor.

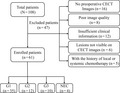

## 1 INTRODUCTION

Neuroendocrine tumors (NETs) are rare types of tumors caused by neuroendocrine cells and can occur in any organ.^1^ The most common location is the gastrointestinal tract (54.5%).^2^ Although rectal NETs (R‐NETs) make up only 1%–2% of all rectal tumors, they account for up to 26.3% of gastroenteropancreatic (GEP) NETs,^3^ and the incidence and prevalence of R‐NETs has been increasing in the last two decades maybe due to the widespread use of screening colonoscopy.^4^, ^5^, ^6^ The growth rate of NETs always presents a relatively indolent feature and the clinical manifestations are mainly the typical carcinoid syndrome.^7^ However, a large proportion has a certain malignant potential and exhibits an aggressive behavior, especially at higher histological grades.^8^, ^9^ It is essential to discriminate low‐grade (G1), intermediate‐grade (G2), and high‐grade (G3) R‐NETs, because the treatment decisions and postoperative management are totally different.^10^ Generally, G1 tumors <20 mm with a low mitotic rate and localized to the submucosa can mostly be removed by local resection.^11^ With higher grades of rectal NET, surgical excision of the rectum and clearance of pelvic nodes are required.^12^

According to the differentiation of tumor cells, gastrointestinal neuroendocrine neoplasms (NENs) are divided into well‐differentiated neuroendocrine tumors (NETs) and poorly differentiated neuroendocrine carcinomas (NECs). Based on the mitotic rate and Ki‐67 proliferation index, NETs are further graded into low‐, intermediate‐, and high‐grade (G1, G2, and G3) NETs.^13^, ^14^ Mitosis rate and Ki‐67 proliferation index usually come from preoperative fine needle aspiration biopsy or postoperative specimens. They are still challenging due to invasiveness in clinical practice and are not accurate enough due to selection bias. In addition, it is difficult to histologically identify the less or more aggressive tumors, the sole criterion for malignancy is determined by metastasis or spread to adjacent organs.^15^ Therefore, the noninvasive and accurate method to preoperatively predict the pathological grade of R‐NETs is urgently needed.

Contrast‐enhanced CT (CECT) can reflect the biological and physiological characteristics of different organs, and it has become an important method for the diagnosis and evaluation of gastrointestinal diseases, especially in colorectal tumors.^16^, ^17^, ^18^ R‐NET usually presents as small isolated nodules, large polyps or mural masses,^19^, ^20^ and early arterial enhancement on CECT.^21^ However, it may only apply information on size, location, shape, and enhancement pattern without spatial information for the entire tumor, and it is difficult to use for tumor pathological grading due to tumor heterogeneity.

CT histogram analysis of cancer images is a noninvasive and emerging method to provide objectively quantified assessment of tumor heterogeneity by analyzing the relationship and distribution of pixel or voxel gray levels in the image. It can apply specific quantitative parameters to identity different tumors or predict pathological grade.^22^ CT histogram analysis has demonstrated good identification and predictive capabilities in a variety of lesions in recent years, such as differentiating benign from malignant or predicting pathological grade.^23^, ^24^ There have been many studies on pancreatic NETs in the past few years.^25^, ^26^ However, to the best of our knowledge, there is almost no literature report on the application of CT histogram analysis in R‐NETs due to the low incidence.

Therefore, we urged to apply and validate CT histogram analysis derived from CECT arterial and venous phase images to explore the optimal parameters for predicting the WHO grade of R‐NETs.

## 2 MATERIALS AND METHODS

### 2.1 Subjects

The Institutional Review Board of our hospital approved this retrospective study and the requirement for patient informed consent was waived. Our pathology database was retrospectively analyzed to identify 108 surgical patients who were confirmed as R‐NET from January 2014 to May 2019. Sixteen patients (including 9 G1, 5 G2, and 2 G3) were excluded due to the absence of preoperative CECT images in the picture archiving and communication system (PACS). Two radiologists with 7 and 17 years of experience in abdominal imaging analyzed all images and excluded another 14 patients and the discrepancies in the image analysis process between the 2 radiologists were resolved by consensus.

**FIGURE 1:**
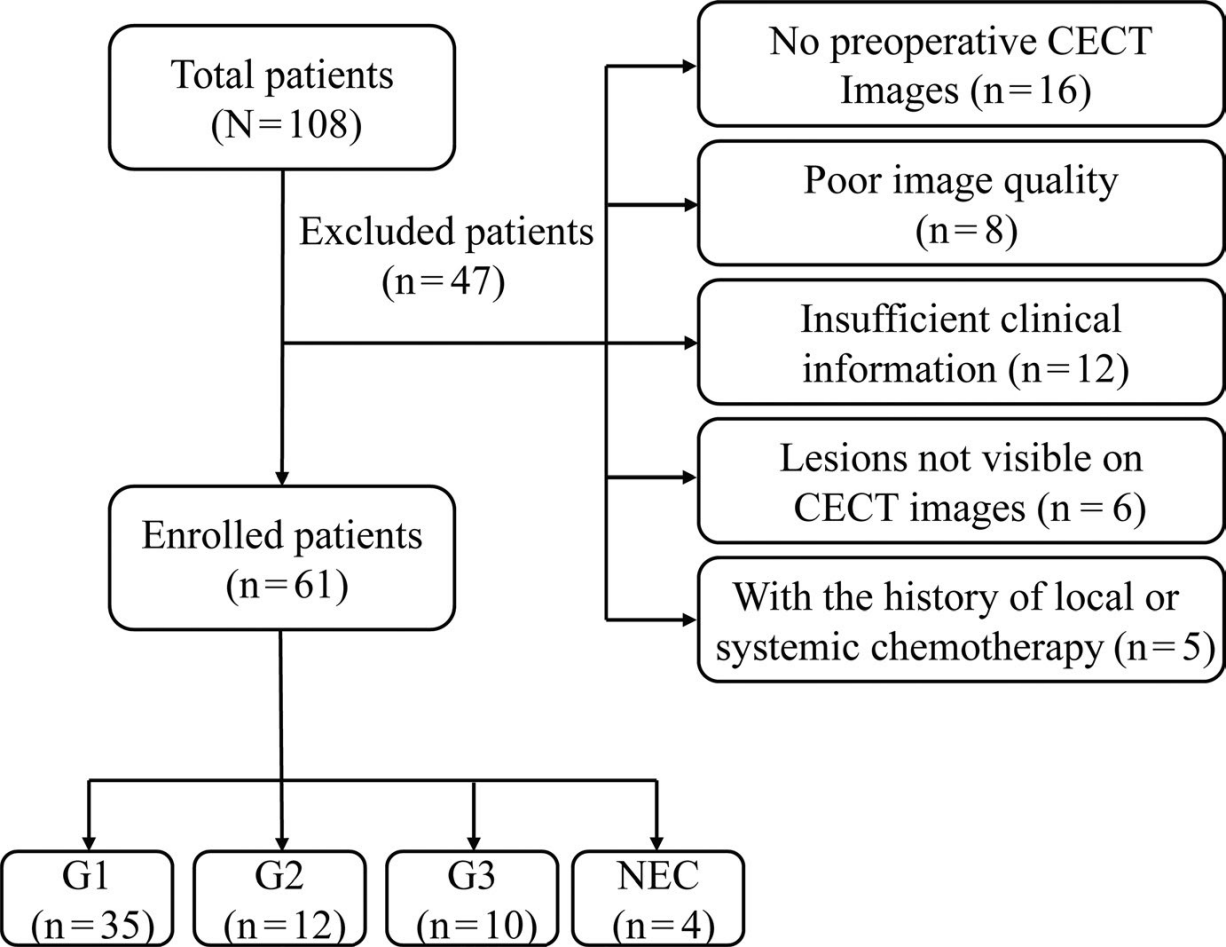
Flowchart of the study population

The exclusion criteria are as follows: (1) no preoperative CECT images (n = 16); (2) poor image quality (n = 8); (3) insufficient clinical information (n = 12); (4) lesions not visible on CT images (n = 6); and (5) with the history of local or systemic chemotherapy (n = 5). Finally, 61 patients were involved in this retrospective study (32 males, 29 females; mean 49.26 ± 11.36 age years; range, 20–74 years) and the inclusion criteria are as follows: (1) histopathologically confirmed R‐NETs at surgery; (2) included preoperative CECT examination; and (3) patients who have not undergone any therapy before surgery. The number of patients were excluded and reasons for exclusion are detailed in Figure 1.

### 2.2 Imaging technique

All patients underwent CECT examinations using a 64‐slice detector scanner (Discovery CT750 HD, GE Healthcare, USA) before surgery. The scan range, which is from the upper edge of the liver to the lower edge of the pubic symphysis, covered the whole abdomen with feet‐first position and all patients were instructed to fast for 4 hours before the examination. Each patient was administered with the iopromide contrast medium (Ultravist 370, 370 mg/ml, Bayer Schering Pharma, Berlin, Germany) in an antecubital vein of the right arm with an 18‐gauge intravenous access using a power injector (Stellant, Medrad, CO, USA) at a rate of 3.5 ml/s and the dose of contrast medium was 1.5 ml/kg body weight, followed by 20 ml saline solution. The arterial phase was initiated 5–8 seconds after the bolus‐tracking threshold of 120 HU was reached at the abdominal aorta and the venous phase began at the 25–30 s after the accomplishment of arterial phase imaging. Optimal delay of arterial phase and venous phase were performed between 25–30 s and 55–60 s, respectively, with the bolus‐tracking technique. Imaging parameters of CECT were as follows: tube voltage, 120 kV; tube current, 120–250 mA; beam collimation, 64 × 0.625; rotation time, 0.5 s; detector pitch, 0.984; matrix, 512 × 512; slice thickness, 5 mm; reconstruction interval of 0.625 mm; and the scan time, 6–8 seconds. Multi‐planar reformatted images with a slice thickness of 1 mm and a slice interval of 0.625 mm in all patients and the thin‐sliced reconstructed images are more sensitive to the detection of small lesions.

### 2.3 Imaging analysis

Without the knowledge of clinical information and pathological findings, two radiologists with 7 and 17 years of abdominal imaging experience evaluated CT imaging features on our hospital RIS system. These features including the following information: (1) the size of tumor; (2) the primary site of tumor, which is divided into low (<5 cm), medium (5–10 cm), and high (10–15 cm) according to the distance of the tumor from the anal margin; (3) enhancement pattern of arterial phase (hypervascular or hypovascular); (4) CT‐reported lymph node (LN) status (LN‐negative or LN‐positive); and (5) tumor margins (well or ill). The discrepancies in the image analysis process between the two radiologists were resolved by consensus.

All raw CT images of patients were transferred from PACS to a personal computer and radiologists applied developed software (Fire Voxel, New York University, New York, USA) to obtain the CT histogram parameters on arterial and venous phases. Radiologists analyzed and manually draw the region of interest (ROI) without knowing the clinical information and pathological results. Freehand ROI were along the contour of the lesion and the edge of the ROI is 1 mm from the edge of the lesion to avoid artifacts, major vessels, and normal tissue and the highest or lowest slices of the lesions were also excluded to reduce the measurement error. Finally, each layer of ROI is automatically fused into a whole tumor volume of interest (VOI) and each merged VOI automatically corresponds to various first‐order parameters in FireVoxel software and these parameters including mean, median, 5th, 10th, 25th, 75th, 90th percentiles, kurtosis, skewness, and entropy.

### 2.4 Clinical data and pathological evaluation

Another two students majored in abdominal imaging collected clinical data and pathological findings in our hospital RIS system. Clinical data were as follows: sex, age, and carcinoembryonic antigen (CEA) level. The routine blood tests for testing CEA level were within 1 week before CT examination and the level of CEA was divided into two groups according to the standards of our hospital: normal group (<=5 ng/ml) or exception group (>5 ng/ml). We invited pathologist (Duan Y) to evaluate all pathological data again based on the 2017 WHO classification criteria. Finally, we recorded the grade of R‐NETs and the Ki‐67 index according to the new assessment results.

### 2.5 Statistical analysis

Statistical analysis was performed with SPSS version 22 (Chicago, IL) and MedCalc (MedCalc Software, Mariakerke, Belgium). All tests were two‐sided and values of *p* < 0.05 were considered statistically significant. Normality was assessed using the Shapiro–Wilk test (*p* ≥ 0.05 indicates normal distribution). Quantitative data were displayed as means ±SD or median. One‐way analysis of variance was employed to compare the differences of various clinical data and parameters among three groups. If a significant difference was found, the two relevant groups were further compared using the Least‐Significant Difference (LSD) test. The interobserver agreements in relation to CT histogram analysis parameters were verified by intraclass correlation coefficient (ICC) test (excellent agreement, 0.81–1.00; moderate agreement, 0.61–0.80; fair agreement, 0.21–0.40; and poor agreement, 0.00–0.20). Receiver operating characteristic curve (ROC) analysis was used to determine the optimal threshold of each parameter and compare the diagnostic performance of significant CT histogram parameters for identifying G1 from higher grade (G2/G3/NECs) by calculating the area under the ROC curve (AUC), sensitivity, and specificity.

## 3 RESULTS

### 3.1 Clinical and imaging characteristics

Sixty‐one patients were included in the statistical analysis and the clinical and imaging characteristics were showed in Table 1. There were significant differences in tumor size among three groups (*p* = 0.000) and the size of G3/NECs group was significantly larger than G1 group or G2 group. The CEA levels among three groups were significantly different (*p* = 0.029), however, the levels of CEA in three groups were mostly under the normal value and there was no significant difference between G1 group and G2 group (*p* = 0.893). We recorded LN enlargement when the short axis of LNs measured larger than 10 mm and LN status on CT‐reported in three groups were significantly different (*p* = 0.000) that the LN status of most G1 (82.86%) were negative, however, most G2/3/NECs (80.77%) were positive. There was no significant difference in sex, age, and the location of the tumor (*p* = 0.248, 0.098, 0.477, respectively; Table 1). The location of the tumor was divided into low (<5 cm), medium (5–10 cm), and high (>10 cm) according to the distance from the anal verge and the most lesions were located in the lower and middle segments of the rectum, which were below the peritoneal fold line.

**TABLE 1:** Clinical and imaging characteristics.

Characteristics	G1 (n = 35)	G2 (n = 12)	G3&NEC (n = 14)	*p* value
Sex				0.248
Man	17 (48.57%)	5 (41.67%)	10 (71.43%)	
Woman	18 (51.43%)	7 (58.33%)	4 (28.57%)	
Age (y)	46.57 ± 11.98	53.17 ± 8.94	52.64 ± 10.34	0.098
CEA (ng/ml)	1.83 ± 0.90	1.89 ± 1.02	3.00 ± 2.36	**0.029**
Normal	34 (97.14%)	12 (100%)	11 (78.57%)	
Abnormal	1 (2.86%)	0 (0%)	3 (21.43%)	
Size (mm)	8.63 ± 6.85	19.08 ± 8.81	37.86 ± 16.67	**0.000**
<10	26 (74.29%)	2 (16.66%)	2 (14.28%)	
10–19	5 (14.28%)	5 (41.67%)	6 (42.86%)	
>19	4 (11.43%)	5 (41.67%)	6 (42.86%)	
Location				0.477
Low (<5 cm)	14 (40%)	6 (50%)	8 (57.14%)	
Medium (5–10 cm)	17 (48.57%)	5 (41.67%)	3 (21.43%)	
High (10–15 cm)	4 (11.43%)	1 (8.33%)	3 (21.43%)	
CT‐reported LN status				**0.000**
LN‐negative	29 (82.86%)	4 (33.33%)	1 (7.14%)	
LN‐positive	6 (17.14%)	8 (66.67%)	13 (92.86%)	

*P* < 0.05 (bold) indicated that the difference was statistically significant.

Abbreviations: CEA, carcinoembryonic antigen; LN, lymph node; NEC, neuroendocrine carcinoma.

### 3.2 Interobserver agreement

The interobserver agreements calculated by appropriate statistical methods demonstrated excellent repeatability (ICCs ranged from 0.876 to 0.991) for all parameters. Therefore, we randomly selected a measurement result from two radiologists for the data analysis. The ICC values for each CTTA parameters were shown in Table 2.

**TABLE 2:** The interobserver agreement for different histogram parameters between two radiologists.

Parameters	ICC	95% CI
Mean	0.991	0.986‐0.993
Median	0.990	0.986‐0.993
10th percentile	0.941	0.915‐0.958
25th percentile	0.944	0.920‐0.961
75th percentile	0.981	0.972‐0.987
90th percentile	0.976	0.965‐0.983
Skewness	0.938	0.912‐0.957
Kurtosis	0.917	0.881‐0.942
Entropy	0.876	0.822‐0.913

Abbreviations: CI, confidence intervals; ICC, intraclass correlation coefficient.

### 3.3 Comparison of histogram parameters between G1 and HG

**FIGURE 2:**
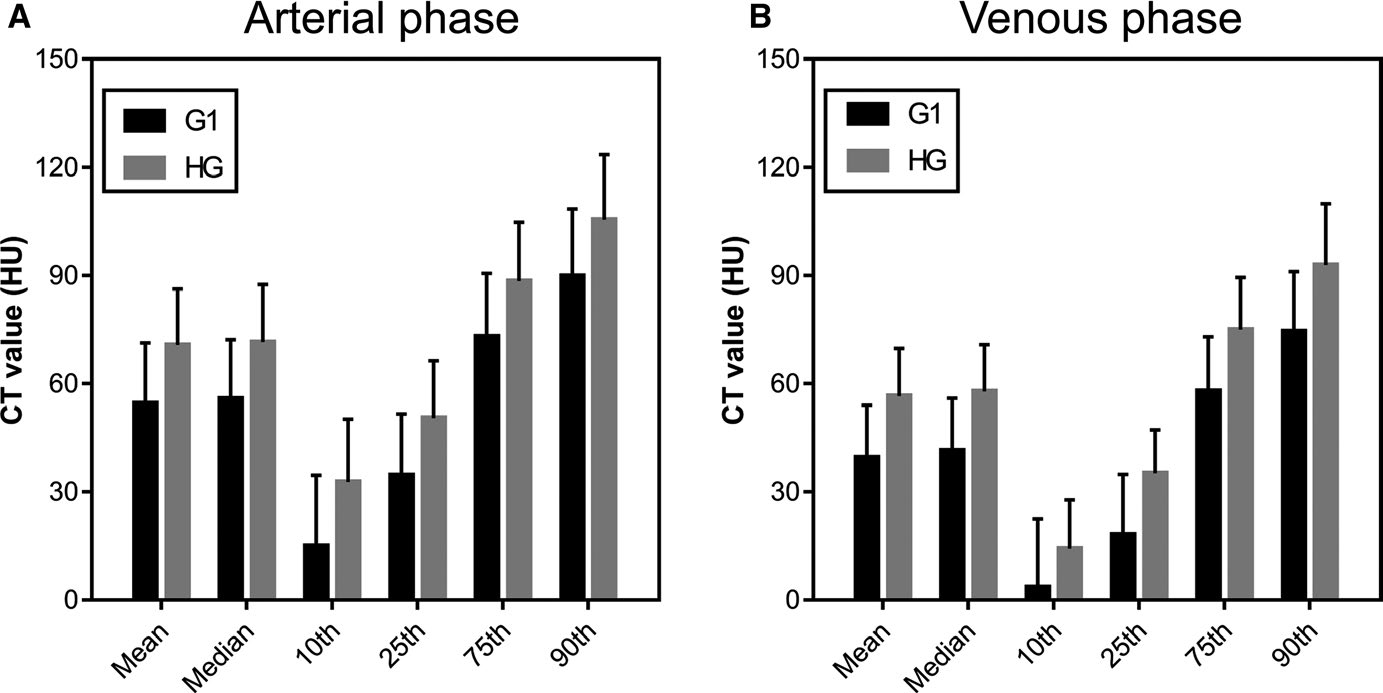
Bar charts showed the comparison of CT histogram parameters for arterial phase (A) and venous phase (B) between G1 and higher grade (HG)

The values of mean, median, 10th, 25th, 75th, and 90th percentiles were significantly lower in G1 group than HG group on arterial and venous phases (all *p* < 0.001). The results were presented in Figure 2 and Table 3. Skewness, kurtosis, and entropy on arterial phase images showed no significant differences between G1 and HG (*p* = 0.853, 0.512, 0.557, respectively). Entropy on venous phase images was significantly different (*p* = 0.017) between G1 and HG, however, skewness and kurtosis showed no significant differences (*p* = 0.654, 0.172, respectively).

**TABLE 3:** Comparison of CT histogram parameters between G1 and higher grade.

Parameters	Arterial phase			Venous phase		
	G1	HG	*p*	G1	HG	*p*
Mean	54.57 ± 16.78	70.77 ± 15.54	<0.001	39.55 ± 14.53	56.54 ± 13.31	<0.001
Median	55.83 ± 16.43	71.62 ± 15.89	<0.001	41.40 ± 14.59	57.81 ± 13.04	<0.001
10th	15.05 ± 19.54	32.77 ± 17.42	<0.001	−3.59 ± 18.87	14.34 ± 13.41	<0.001
25th	34.57 ± 17.09	50.50 ± 15.88	<0.001	18.23 ± 16.62	35.19 ± 12.06	<0.001
75th	73.09 ± 17.46	88.48 ± 16.27	<0.001	57.91 ± 15.16	75.08 ± 14.38	<0.001
90th	89.86 ± 18.63	105.54 ± 17.97	<0.001	74.54 ± 16.53	92.92 ± 16.99	<0.001
Skewness	−0.93 ± 2.02	−1.03 ± 2.17	0.853	−0.93 ± 0.95	−1.06 ± 1.42	0.654
Kurtosis	5.19 ± 18.79	2.68 ± 5.46	0.512	5.38 ± 9.69	9.96 ± 16.09	0.172
Entropy	3.79 ± 0.41	3.73 ± 0.44	0.557	3.57 ± 0.37	3.87 ± 0.58	0.017

Note:

10th, 25th, 75th, and 90th represented 10th percentile, 25th percentile, 75th percentile, and 90th percentile of CT attenuation value of histogram distribution, respectively.

HG, higher grade, including G2, G3, and NEC.

### 3.4 Diagnostic performance of histogram parameters for identifying G1 from HG by ROC analysis

**FIGURE 3:**
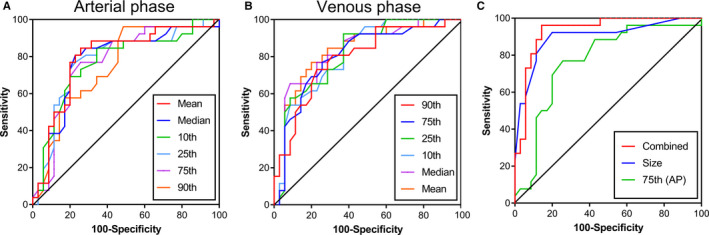
ROC curves for CT histogram parameters in differentiating G1 from higher grade (HG) at arterial phase (A) and venous phase (B) of enhanced CT scan. Combined the size of tumor and the 75th Percentile generated the highest AUC (C)

**FIGURE 4:**
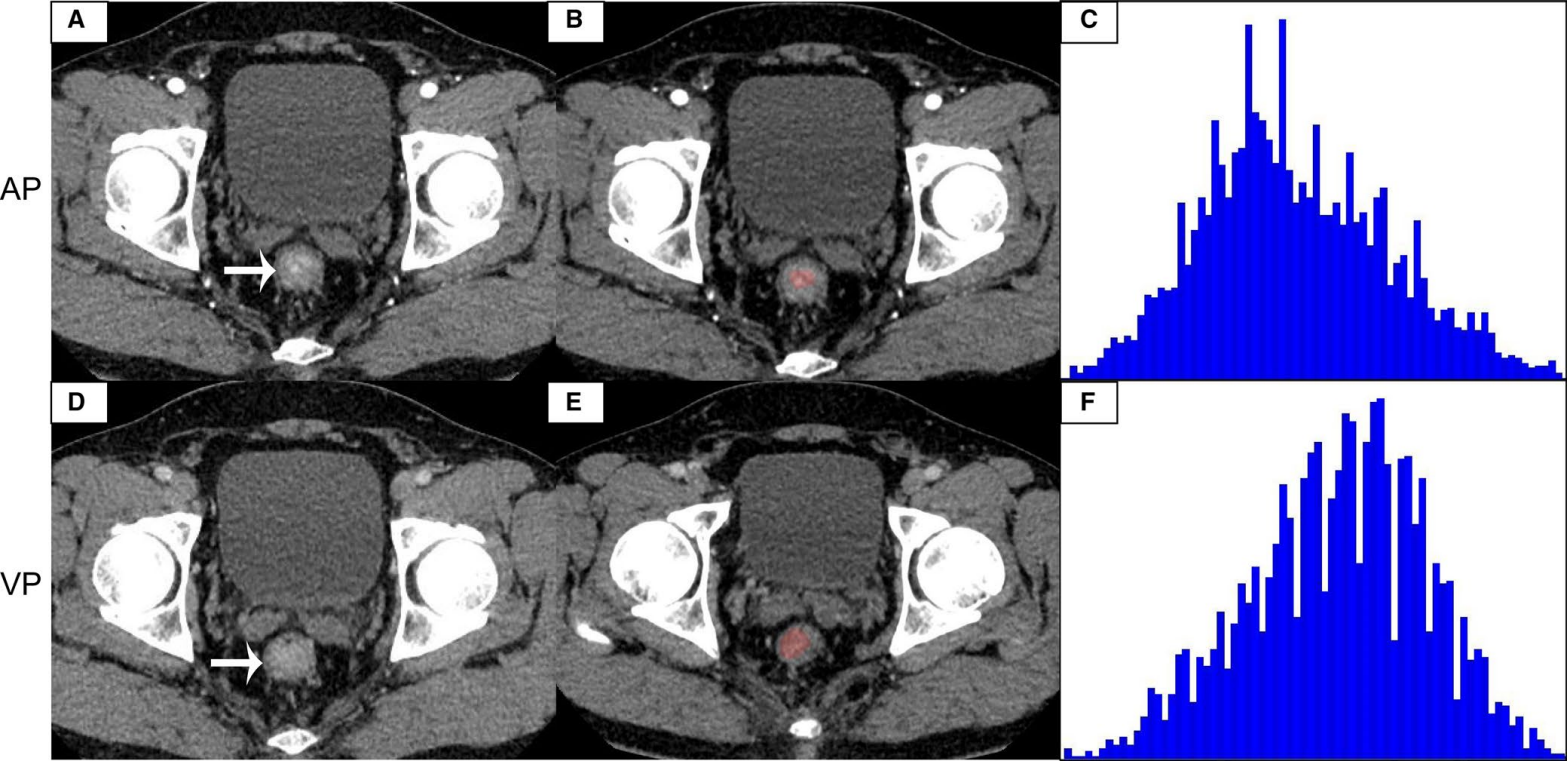
A 51‐year‐old male with G1 R‐NETs. The tumor was significantly enhanced on the arterial phase image (A), and the degree of enhancement is reduced on the venous phase image (D). The tumor region of interest (ROI) was localized on axial CT images at arterial phase (B) and venous phase (E). CT histogram at arterial phase (C) and venous phase (F) were shown

**FIGURE 5:**
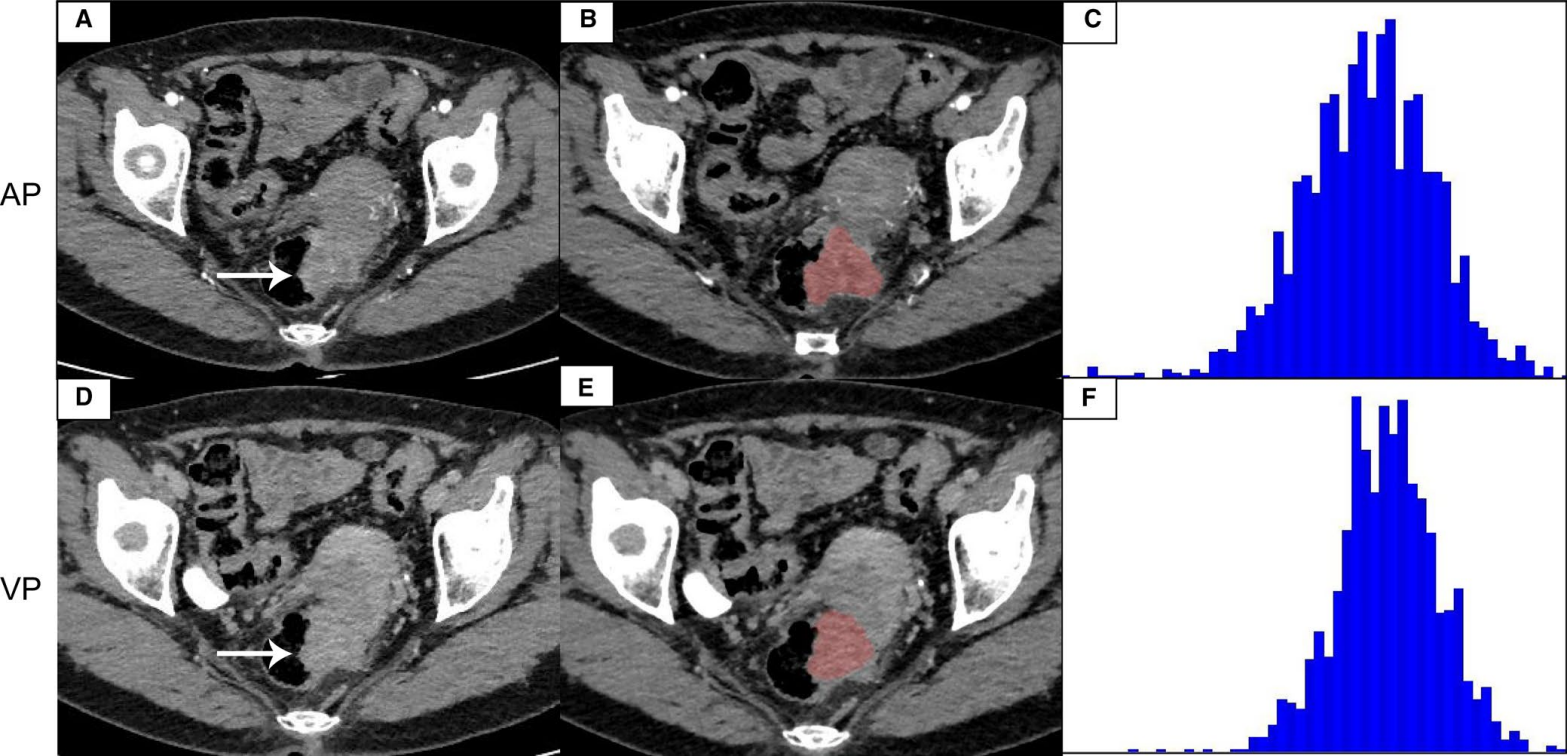
A 54‐year‐old female with G2 R‐NETs. The tumor was significantly enhanced on the arterial phase image (A), and the degree of enhancement is reduced on the venous phase image (D). The tumor region of interest (ROI) was localized on axial CT images at arterial phase (B) and venous phase (E). CT histogram at arterial phase (C) and venous phase (F) were shown.

**FIGURE 6:**
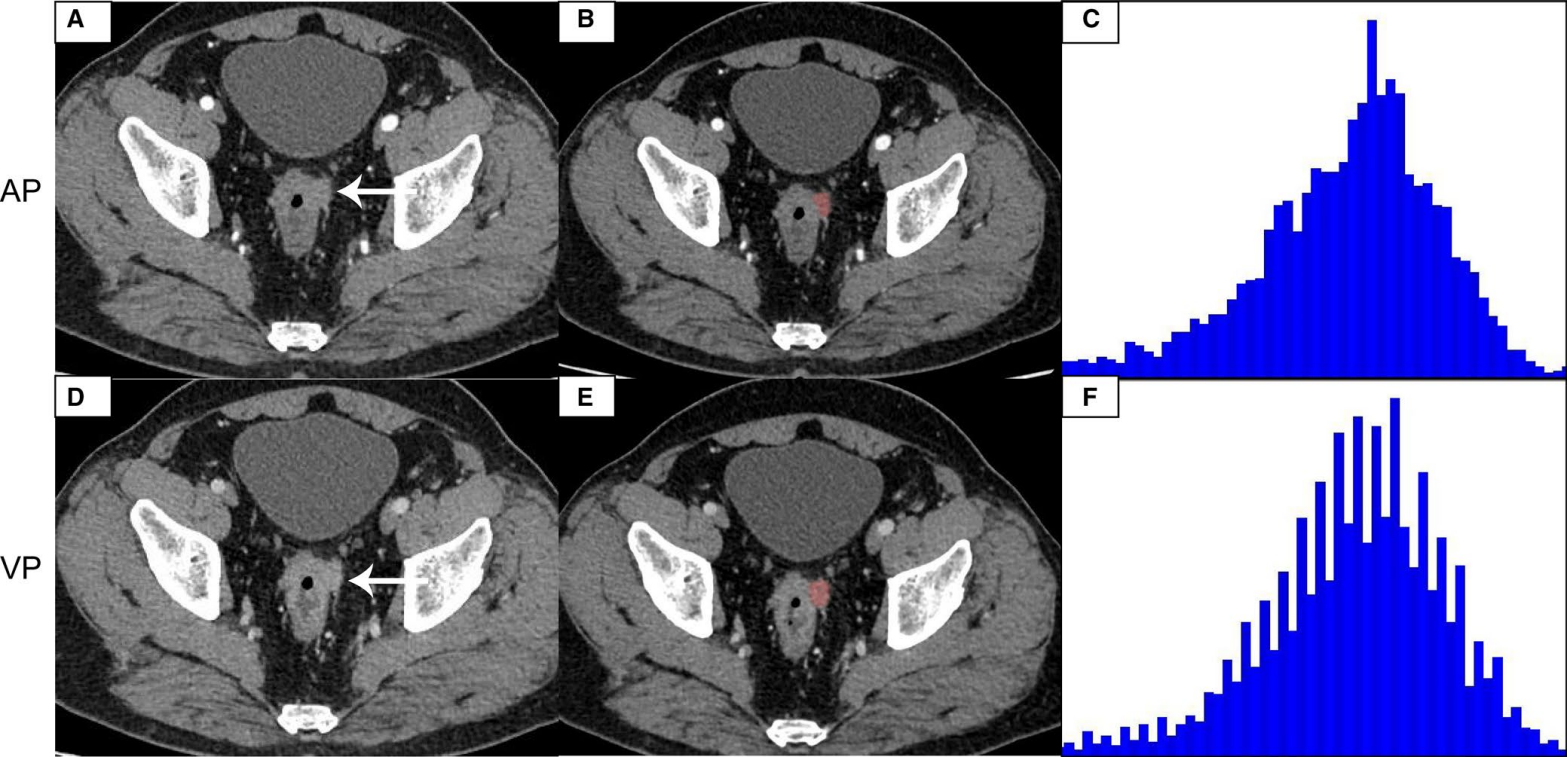
A 56‐year‐old male with G3 R‐NETs. The tumor was significantly enhanced on the arterial phase image (A), and the degree of enhancement is reduced on the venous phase image (D). The tumor region of interest (ROI) was localized on axial CT images at arterial phase (B) and venous phase (E). CT histogram at arterial phase (C) and venous phase (F) were shown

In the ROC analysis, the 75th percentile generated highest AUC (AUC = 0.871, 95% CI, 0.741–0.951) in all CECT histogram parameters for differentiating G1 from HG with sensitivity of 100% and specificity of 74.29%. The size of the tumor also generated high AUC (AUC = 0.899. 95% CI, 0.795–0.962) with sensitivity of 92.31% and specificity of 80.00%. We found that when we combined the 75th percentile and the size of the tumor to diagnose, the maximum AUC (AUC = 0.932, 95% CI, 0.837–0.981, sensitivity, 96.15, specificity, 85.71) was obtained. The ROC curves analysis showed in Figure 3. The AUC of skewness, kurtosis, and entropy were both lower than 0.6 and the diagnostic performance of histogram parameters were presented in Table 4. Representative images of G1, G2, and G3 R‐NETs, a flow diagram of delineating ROIs, and the results of histogram analysis are shown in Figures 4, 5, 6, respectively.

**TABLE 4:** Diagnostic performance of histogram parameters for differentiating G1 from higher grade.

Parameters	AUC (95% CI)	Cut‐off	Sensitivity (%)	Specificity (%)	You‐Index
Mean (AP)	0.792 (0.669‐0.886)	60.81	84.62	74.29	0.589
Median (AP)	0.777 (0.653‐0.874)	63	80.77	77.14	0.579
10th (AP)	0.840 (0.704‐0.931)	22	91.67	65.71	0.574
25th (AP)	0.864 (0.733‐0.947)	44	83.33	80.00	0.633
75th (AP)	0.871 (0.741‐0.951)	79	100	74.29	0.743
90th (AP)	0.833 (0.696‐0.926)	103	75.00	82.86	0.579
Mean (VP)	0.864 (0.733‐0.947)	45.90	91.67	71.43	0.631
Median (VP)	0.864 (0.733‐0.947)	42	100	62.90	0.629
10th (VP)	0.838 (0.702‐0.929)	13	66.67	91.43	0.581
25th (VP)	0.840 (0.704‐0.931)	21	100	62.86	0.629
75th (VP)	0.851 (0.717‐0.938)	56	100	60.00	0.600
90th (VP)	0.819 (0.679‐0.916)	82	83.33	71.43	0.548
Entropy (VP)	0.576 (0.423‐0.719)	3.95	33.33	94.29	0.276
Size (mm)	0.899 (0.795‐0.962)	12	92.31	80.00	0.723
Combined	0.932 (0.837‐0.981)	NA	96.15	85.71	0.819

Note:

10th, 25th, 75th, and 90th represented 10th percentile, 25th percentile, 75th percentile, and 90th percentile of CT attenuation value of histogram distribution, respectively.

AUC, area under the receiver operating characteristic curve; CI, confidence intervals.

NA, not application; and Combined, combined the size of the tumor and the 75th percentile.

## 4 DISCUSSION

This single‐center retrospective study focused on the preoperative predictive performance of CECT histogram parameters for the histologic grade of R‐NETs on the arterial and venous CT images. Generally, the treatment and the clinical management are different between patients of different pathological grades due to the grade of R‐NETs is a risk factor for recurrence.^27^ Grade 1 R‐NETs can be resected endoscopically while grades 2 or 3 R‐NETs are more suitable for surgical resection. Grades 1 or 2 R‐NETs are followed up annually and at 4–6 months for grade 3 R‐NETs during the first year and then, annually. The results of this study showed an excellent potential for distinguishing G1 R‐NETs from higher grade (G2/3 R‐NETs and NECs), which played an important role in clinical decision making and patient management. In this study, the clinical and CT characteristics were analyzed. The most relevant clinical factor with histopathological grade was the size of the tumor that the G1 R‐NETs were mostly below 10 mm and smaller than the G2/3 /NECs and previous findings had proven the tumor size was associated with the survival and the metastases.^28^, ^29^

According to the research of Ushigome et al,^30^ it is considered difficult to predict LN metastasis on CT in patients with R‐NETs. However, CT‐reported LN status was significantly different in the three groups in our study, with most being negative in the G1 group and mostly positive in the G3 group. The results are similar to previous studies, which showed that the higher the G grade, the higher the risk of regional LN metastasis.

Previous study demonstrated that male gender was a risk factor of R‐NETs and it predominantly occurred during the sixth decade of age and the tumor mostly located in the lower and intermediate portion of the rectum.^31^ The location of the tumor in our study was consistent with it. However, there was no significant difference in gender and the average age was 49.26 ± 11.36 years in our study. This may be due to the small size of our research, or due to the differences between East and West races.

Histogram analysis is a rapidly emerging and noninvasive method in the field of medical imaging. It can objectively and quantitatively evaluate tumor heterogeneity, regularity, and roughness of images by evaluating the distribution of voxel gray levels without requiring additional invasive procedures. Mean value of contrast CT histogram often reflects the average enhancement degree of the whole lesion. Azoulay et al^32^ indicated that high mean value was found to be related with high tumor grade in pancreatic neuroendocrine tumor and this was similar with our study that the mean value of G1 group were significantly lower than G2/G3/NECs. When the data are similar to a normal distribution, the median is capable to the mean. In our study, the values of these two parameters are roughly the same and the ability to distinguish G1 group from G2/3/NEC is also comparable, but the AUC of mean is slightly larger than the median.

Due to tumor heterogeneity, morphological characteristics (such as mitotic rate, necrosis, and cell polymorphism) are different between different pathological levels or benign and malignant tumors.^33^ The lower percentiles (10th and 25th) usually reflected the fat components, cystic components, foci of hypoxia, and micronecrosis in the lesions due to the rapid tumor cell growth. In our study, the lower percentiles of G1 group were lower than G2/3/NEC groups which was in contrast to previous research,^25^ which may be caused by the fatty changes in the lesions or be influenced by the intestinal gas. The higher percentiles (75th and 90th) reflect the tumor angiogenesis and the blood perfusion. R‐NETs always presented as hypervascular and a well‐circumscribed solid mass that significantly enhanced in arterial phase images.^34^ The higher percentiles of G2/3/NEC tumors were significantly larger than G1 tumor and this result was similar to the previous research.^35^

Generally speaking, tumors with increased histological heterogeneity may have higher entropy, higher kurtosis, and positive skewness.^22^ Skewness is a measure of the asymmetry in the distribution of pixel densities. Positive skewness indicates that the tail on the right side of the histogram is longer than the left side. Kurtosis is associated with the peakedness of CT value distribution in the lesions and a positive or negative kurtosis means that the pixel distribution curve is either more or less peaked than a normal distribution curve. Previous study demonstrated G2/G3 P‐NETs showed higher skewness and higher kurtosis than G1 P‐NETs.^36^, ^37^ Skewness and kurtosis showed no significant differences between G1 and G2/3/NECs in our study. The discrepancies may be caused by differences in tumor microenvironment between P‐NETs and R‐NETs, although they were all derived from neuroendocrine cells.

Entropy is a measurement of random irregularity of the histogram and the higher intra‐tumoral heterogeneity with the higher entropy. The previous study reported that the differences in entropy may be useful to differentiate benign lesions from malignant tumors and the higher entropy indicated higher tumor malignancy.^38^ Lotfalizadeh et al reported that higher grade tumors presented more heterogeneous than lower grade tumors on contrast‐enhanced imaging.^39^ In our study, the entropy of G2/G3/NECs groups on venous phase was higher than the G1 group and this may be hemorrhage, increased cellularity density, and the formation of fiber structure.

There were several limitations of our study. First, the number of patients included in this study was small and this was a single‐center retrospective study. We need to include more cases in different institutions to obtain more reliable and stable results. Second, we did not separate NECs into groups due to the small cases. Finally, we just apply first‐order parameters to evaluate the WHO grade of R‐NETs and we would like to apply higher‐order parameters or radiomics in the future study.

In conclusion, CECT histogram analysis including arterial and venous phase images may be a promising and noninvasive method to differentiate G1 from G2/3/NECs tumors. Combination of histogram analysis and the size of the tumor could help to differentiate the grade of R‐NETs more accurately. These results can play an important role in clinical decision making and patient management.

## CONFLICT OF INTEREST

The authors of this manuscript declare that there is no conflict of interest that could be perceived as prejudicing the impartiality of the research reported.
